# Comparison of Proteomics Profiles of *Campylobacter jejuni* Strain Bf under Microaerobic and Aerobic Conditions

**DOI:** 10.3389/fmicb.2016.01596

**Published:** 2016-10-13

**Authors:** Ramila C. Rodrigues, Nabila Haddad, Didier Chevret, Jean-Michel Cappelier, Odile Tresse

**Affiliations:** ^1^LUNAM Université, Oniris, Université de NantesNantes, France; ^2^INRA, UMR 1014 SECALIMNantes, France; ^3^INRA UMR 1319 MICALIS, PAPPSOJouy-en-Josas, France

**Keywords:** foodborne pathogen, *Campylobacter jejuni*, proteomics, aerotolerance, oxidative stress

## Abstract

*Campylobacter jejuni* accounts for one of the leading causes of foodborne bacterial enteritis in humans. Despite being considered an obligate microaerobic microorganism, *C. jejuni* is regularly exposed to oxidative stress. However, its adaptive strategies to survive the atmospheric oxygen level during transmission to humans remain unclear. Recently, the clinical *C. jejuni* strain Bf was singled out for its unexpected ability to grow under ambient atmosphere. Here, we aimed to understand better the biological mechanisms underlying its atypical aerotolerance trait using two-dimensional protein electrophoresis, gene expression, and enzymatic activities. Forty-seven proteins were identified with a significantly different abundance between cultivation under microaerobic and aerobic conditions. The over-expressed proteins in aerobiosis belonged mainly to the oxidative stress response, enzymes of the tricarboxylic acid cycle, iron uptake, and regulation, and amino acid uptake when compared to microaerobic conditions. The higher abundance of proteins related to oxidative stress was correlated to dramatically higher transcript levels of the corresponding encoding genes in aerobic conditions compared to microaerobic conditions. In addition, a higher catalase-equivalent activity in strain Bf was observed. Despite the restricted catabolic capacities of *C. jejuni*, this study reveals that strain Bf is equipped to withstand oxidative stress. This ability could contribute to emergence and persistence of particular strains of *C. jejuni* throughout food processing or macrophage attack during human infection.

## Introduction

*Campylobacter*, a Gram-negative spiral-shaped bacterium, is currently recognized as the main cause of foodborne gastroenteritis in humans in developed countries (Epps et al., 2013; Gölz et al., 2014). In 2014, this microorganism was responsible for more than 236,000 confirmed campylobacteriosis cases in Europe, with an incidence of 71 per 100,000 people (EFSA and ECDC, 2016). This incidence increased by 9.6% compared with 2013. Of these reported cases, 81.8% were related to *C. jejuni*, 7.1% to *C. coli*, 0.13% to *C. lari*, 0.09% to *C. fetus* and 0.07% to *C. upsaliensis* (EFSA and ECDC, 2016). *C. jejuni* infection induces acute gastroenteritis and is marked by fever, diarrhea, and intense abdominal pains. The infectious dose was estimated to be as low as 500–800 bacteria (Robinson, 1981; Young et al., 2007). In addition, some patients can develop post-infection complications including Guillian Barré Syndrome (GBS) and its variant form Miller Fisher Syndrome (MFS), a chronic and potentially fatal form of paralysis (Nachamkin, 2002; Kuwabara, 2011; Huizinga et al., 2015).

Overall, as an obligate microaerobic microorganism, *C. jejuni* requires 5–10% oxygen for its optimal growth and cannot grow under atmospheric oxygen levels (Kaakoush et al., 2007; Macé et al., 2015). However, it is regularly exposed to oxidative stress during its life cycle. During respiration, the reduction of dioxygen to H_2_O is accompanied by the production of a variety of toxic intermediate products called reactive oxygen species (ROS) including the superoxide anion radical (${\text{O}}_{2}^{•-}$), hydrogen peroxide (H_2_O_2_) and the hydroxyl radical (^•^OH), which can cause irreversible damage to nucleic acids, proteins, and lipids (Cabiscol et al., 2000). To detoxify ROS, *C. jejuni* produces the main oxidative stress defense enzymes: superoxide dismutase (SodB), catalase (KatA), and alkyl hydroperoxide reductase (AhpC) (Pesci et al., 1994; Grant and Park, 1995; Baillon et al., 1999; Atack and Kelly, 2009). In comparison with other enteric bacteria, *C. jejuni* appears to have very limited capacity to regulate gene expression in response to environmental stresses, especially oxidative stress (Park, 2002). The key regulators of the stress defense systems described in other enteric bacteria are absent in this pathogen. For instance, SoxRS, OxyR, and the sigma factor RpoS, for general stress-defense gene regulation under hostile environmental conditions, are not present in the *C. jejuni* genome (Parkhill et al., 2000). Despite the lack of common bacterial stress responses and the fragility of this organism faced with atmospheric oxygen concentrations, it is surprising that *C. jejuni* is the major foodborne bacterial causative agent of gastrointestinal disorders in humans. Its adaptive strategies to survive the atmospheric oxygen level during transmission to humans remain unclear.

The investigation of *C. jejuni* ability to grow *in vitro* under atmospheres with high oxygen tension has not been conducted in depth, although its ability to tolerate and adapt to oxygen has often been reported (Jones et al., 1993; Fields and Thompson, 2008; Sulaeman et al., 2012; Kassem et al., 2014; Rodrigues et al., 2015; Turonova et al., 2015). In our laboratory, we have isolated an atypical clinical *C. jejuni* strain (Bf) characterized by its ability to grow under aerobic conditions (Rodrigues et al., 2015). This was correlated to a lower susceptibility to compounds generating superoxide and peroxide stresses (Rodrigues et al., 2015). The first analyses of the genome of Bf did not reveal any deletion/insertion, SNPs, or organization of the genes already described to be involved in the sub-system of oxygen detoxification in other *C. jejuni* strains (Bronnec et al., 2016).

In this study, the adaptation of Bf to aerobic conditions was investigated by comparing two-dimensional (2-D) profiles of multiplying cells in microaerobic conditions and aerobic conditions. The gene expression of differentially regulated proteins involved in the sub-system of oxygen detoxification and the H_2_O_2_ breakdown enzymatic activity were then compared between cells grown in microaerobic conditions, cells grown in aerobic conditions, or cells acclimated to aerobic conditions to identify the underlying molecular mechanisms contributing to the atypicity of this strain.

## Materials and methods

### Bacterial strains and growth conditions

*C. jejuni* Bf and *C. jejuni* NCTC 11168 were stored at −80°C in brain and heart infusion medium (BHI) with sterile glycerol at 20%. Preparation of *C. jejuni* cells was performed as previously described in Rodrigues et al. (2015) with the following modifications. Before each experiment, the strains were grown on Karmali agar plates (Oxoid, France) at 42°C for 48 h (h) in microaerobic conditions (MAC) generated using gas replacement jars operated by a MACSmics gassing system (BioMérieux, France) with a gas blend composed of 5% O_2_, 10% CO_2_ and 85% N_2_ and four filled/flushed cycles at −50 kPa.

To obtain cells of *C. jejuni* Bf cultured in aerobic conditions (AC) (ambient atmosphere), cells were first subcultured in microaerobic conditions at 42°C on Karmali agar for 48 h and then harvested and resuspended in peptone saline solution. An absorbance at 600 nm (OD_600nm_) of 0.03 (≈ 10^6^ CFU.mL^−1^) was used to standardize cell suspension. The bacterial suspension was then inoculated onto Karmali agar plates and incubated under aerobic conditions for 24 h at 42°C. The aerobically acclimated cells (AAC) were prepared as described before by Rodrigues et al. (2015). Briefly, *C. jejuni* Bf was first subcultured under microaerobic conditions onto Karmali plates for 48 h. Colonies were then harvested and subcultured three times successively on Karmali agar plates followed by incubation at 42°C for 24 h under aerobic conditions.

#### Protein extraction and two-dimensional gel electrophoresis (2-DE)

Proteins of harvested cells of *C. jejuni* Bf were extracted according to Bièche et al. (2012). Briefly, after resuspension in 200 mM glycine solution, followed by 100 mM Tris–HCl pH 7.0 and 10 mM Tris–HCl pH 7.0 solutions, cells were lysed by ultrasonic treatment at 25 kHz. Ultracentrifugation at 188,000 × *g* was conducted to separate cytosoluble proteins from membrane fractions. Then, purified proteins were quantified using the Micro BCA™Protein Assay Kit (Perbio-Science, France) and stored at −80°C.

Concentration of 30 μg of proteins to reach a final volume of 15 ± 5 μL was performed using the Concentrator 5301 (Eppendorf, Le Pecq, France) at room temperature. Then, each sample was resuspended in 185 μL of rehydration buffer as described previously (Haddad et al., 2009; Bièche et al., 2012) and rehydrated using a Protean IEF cell system at 20°C (Bio-Rad, France) overnight in the system with a 50 V current and 11 cm *pI* 4–7 IPG strip (Bio-Rad, France) or *pI* 6-11 IPG strip (GE Healthcare Life Sciences, France). Isoelectrofocusing (IEF) was performed as follows: from 0 V to 250 V for 3 h, from 250 V to 4500 V for 3 h and at 4500 V until 42,000 Vh was reached and at 500 V for 20 h maximum. Finally, strips were equilibrated for 2 × 20 min in 1.5 mL of buffer 1 and 2, respectively (buffer 1: 6 M urea, 2% SDS, 0.05 M Tris–HCl (pH 8.8), 30% glycerol, and a 0.02% bromophenol blue, supplemented with 2% DTT; buffer 2: 6 M urea, 2% SDS, 0.05 M Tris–HCl (pH 8.8), 30% glycerol, and 0.02% bromophenol blue, supplemented with 4% iodoacetamide (Bio-Rad, France).

The second dimension was performed using a precast polyacrylamide gel gradient (4–15%) (Bio-Rad, France) covered with 1% low-melting point agarose (Bio-Rad, USA) and run at 20 mA/gel at 14°C using the Criterion^™^ Dodeca^™^ Cell (Bio-Rad, France) until the migration of bromophenol blue reached the bottom of the gels. Proteins in gels were silver-stained according to Garnier et al. (2010) to reveal the cytoplasmic proteome map and scanned with a GS-800 densitometer (Bio-Rad, France) operated with the QuantityOne^®;^ software (Bio-Rad, France) at a resolution of 42.3 μm.

### Gel image and statistical treatment

Comparisons between gels (detection, quantification, alignment, spot matching, and statistical analyses) were carried out using Progenesis Samespots® 4.0 software (NonLinear Dynamics, Newcastle upon Tyne, UK). Statistical analysis of protein abundance was carried out on six gels for each condition, obtained from three independent cultures and two technical replicates for each independent culture. Principal Component Analysis (PCA) was performed to highlight differences between the conditions tested. Normalization of the spots was based on the abundance ratio, which was selected in the algorithm developed by Progenesis SameSpots®. Differences between matched spot intensities were statistically confirmed by calculating an ANOVA (at a 5% significance level). *P*-values were refined by the *q*-value (at 5% significance level) to discard any false positives as described before (Bièche et al., 2012). The *q*-value calculates the false discovery rate (FDR) for any spot selected with a *p*-value < 0.05 (Table S1). The power threshold used was 80% (*P* > 0.8) which is considered to be relevant for 3 biological replicates and 3 technical replicates for each condition and only fold of at least 1.5 was defined as significant.

### Protein identification by LC-MS/MS

When spots of interest were located, 2-DE gels were run again using 700 μg of protein, and stained with BioSafe colloidal Coomassie blue (Bio-Rad, France). Spots of interest were excised for further LC-MS/MS analysis using PAPPSO platform facilities (http://pappso.inra.fr). In-gel digestion was performed according to a standard trypsinolysis protocol. Gel plugs were first washed twice with 25 mM ammonium bicarbonate-50% acetonitrile. Proteins were reduced and alkylated prior to digestion. 100 μl of 10 mM DTT was added and the sample was incubated for 1 h at 55°C. The samples were allowed to cool and the supernatant was removed before 100 μL of 55 mM iodoacetamide was added. Gel pieces were washed further with 25 mM NH_4_CO_3_ and dehydrated in acetonitrile. Digestion was conducted for 6 h at 37°C with 100 ng of modified trypsin dissolved in 10 μL of 25 mM NH_4_CO_3_. For further LC-MS/MS analysis, peptides of the digestion mix were extracted from the gel with 50% (v/v) acetonitrile, 0.5% trifluoroacetic acid in water, and then with pure acetonitrile. Both peptide extracts were pooled, dried in a vacuum speed concentrator, and suspended in 25 μL of 2% (v/v) acetonitrile, 0.08% (v/v) trifluoroacetic acid in water. LC-MS/MS analysis was achieved on an Ultimate 3000 LC system (Dionex, Voisins-le-Bretonneux, France) linked to an LTQ-Orbitrap Discovery mass spectrometer (Thermo, USA) via a nanoelectrospray ion source. Samples (4 μL) were loaded at 20 μL/min onto a pre-column cartridge (stationary phase: C18 PepMap 100, 5 μm; column: 100 μm inner diameter, 1 cm in length; Thermo, France) and desalted with 2% acetonitrile −0.1% formic acid (buffer A). After 4 min, the pre-column was connected to the separating nano-column Pepmap C18 (0.075 × 15 cm, 100 Å, 3 μm; Thermo, France) and a linear gradient was started from 2 to 36% of buffer B (80% acetonitrile, 0.1% formic acid) at 450 nl/min over 12 min. The doubly and triply charged precursor ions were subjected to MS/MS fragmentation with a 1 min exclusion window, and with classic peptide fragmentation parameters (Qz = 0.22, activation time = 50 ms, collision energy = 35%). Proteins were identified by querying MS/MS data against the *Campylobacter jejuni* subsp. *jejuni* strain NCTC 11168 protein database (Uniprot, 2015.03.31) along with an in-house contaminant database, using the X!Tandem software (X! tandem CYCLONE (2011.12.01.1), http://www.thegpm.org) with the following parameters: one missed trypsin cleavage, alkylation of cysteine and conditional oxidation of methionine precursor and fragment ion set to 10 ppm and 0.5 Da, respectively. A refined search was added with similar parameters, except that the semi-tryptic peptides, and the possibly N-terminal acetylated proteins were included. All the peptides that matched an *E*-value lower than 0.05 were parsed with an in-house program (http://http:/PAPPSO.inra.fr/bioinformatique.html). Proteins identified by at least two unique peptides and a log (*E*-value) lower than −2.6 were considered to be validated. The KEGG pathway database of *C. jejuni* NCTC 11168 (http://www.genome.jp/kegg/) was used to infer the function and cell location of the identified proteins (Table S1).

### RNA extraction and quantitative RT-PCR

Cell cultures of *C. jejuni* NCTC 11168 and *C. jejuni* Bf strains were grown in microaerobic conditions and *C. jejuni* Bf was cultured in aerobic conditions and aerobically acclimated as described above. Identical number of cell subculturing for all conditions was performed. Bacterial suspension were immerged in RNA Protect Reagent (Qiagen, Hilden) and centrifuged at 3300 × *g* for 6 min at 4°C, then resuspended in 1 mL of Extract-All (Eurobio, France) and 0.2 mL of chloroform. After treating samples with DNase, the quality, and quantity of RNA were checked using a spectrometer NanoDrop (NanoDrop® 2000, Thermo Scientific) and then adjusted to 20 ng/μl. Samples of RNA were submitted to reverse transcription using RevertAid H Minus First-Strand cDNA synthesis kit (Euromedex) and random hexamer primers (Eurobio) according to the manufacturer's instructions and Bièche et al. (2012) with the following modifications: DNA removal was checked by PCR using 341F/758R primers and the gene expression of *kat*A, *sod*B, *ahp*C, *tpx, trxB*, and *rrs* was assessed using specific primers (Table 1). The *rrs* gene was used as the endogenous control as described previously (Li and Schellhorn, 2007; Hyytiäinen et al., 2012). The composition of the PCR mix was as follows: 5.0 μL of sample, reverse primer (1 μM), forward primer (1 μM), and 12.5 μL of SYBR Green I Master Mix. The amplification program included an initial denaturing step at 95°C (10 min), followed by 40 cycles of 95°C (15 s) and 60°C (1 min). A negative control (without cDNA) was included in each run. The relative quantification of the specific gene expression was calculated according to the 2^−ΔΔC*t*^ method. The experiments were performed in triplicate from three independent cultures. Significant differences were determined using Student's *t*-test comparisons at a 0.05 significance level.

**Table 1:** **Primers used in this study**.

**Primers**	**Sequences 5′ → 3′**
*341 F*	CCTACGGGAGGCACGAG
*758 R*	CTACCAGGGTATCTAATCC
*ahpC* F	TTCGTACGGCTGGAGATAAG
*ahpC* R	GTGAAAGAACAGGCGAAGAG
*katA* F	CAAACAGCTATGATAATAGCC
*katA* R	GGAGCATATCTTTGTGCTACG
*sodB* F	CTTCAAACGCAGCTACACCA
*sodB* R	CCCAGTTAATATGAGCATAGAA
*tpx* F	GCCAGTTACAATGGTGCTGA
*tpx* R	TTTGCCACAAAATCACTTGC
*trxB* F	AGCCCAAGTTATGGATGGAA
*trxB* R	TTTGGAGCTGAGCCTGT
*rrs* F	AAGGGCCATGATGACTTGACG
*rrs* R	AGCGCAACCCACGTATTTAG

### Enzymatic activity assays

Catalase-equivalent activity (CEA) was determined according to the methodology proposed by Li and Schellhorn (2007), with the following modifications: sensitivity and accuracy of this method was evaluated using a commercial Bovine liver catalase (Sigma C-30, France) as a quality control. To prepare the standard curve of catalase, bovine liver catalase was diluted with 0.05 M phosphate buffer (pH 7.0) to obtain a catalase quantity ranging from 0.01 to 2.00 units. A volume of 100 μL of catalase solution at different concentrations was added to the quartz cuvette (Hellma, Germany) containing 5 mM H_2_O_2_ diluted in phosphate buffer. Subsequently, the optical density was determined at 240 nm within 5 min. The decomposition rate of H_2_O_2_, which was proportional to the reduction of the absorbance at λ = 240 nm, was used to calculate CEA. CEA represents the H_2_O_2_ breakdown capability of a whole cell lysate sample which is converted to catalase activity based on the calibration curve obtained with the commercial catalase. To measure the CEA, total protein extracts obtained from exponential cultures of *C. jejuni* NCTC 11168 and *C. jejuni* Bf strains grown in microaerobic conditions, *C. jejuni* Bf cultured in aerobic conditions, and *C. jejuni* Bf aerobically acclimated were used. Total proteins were extracted using the Bacterial Protein Extraction Reagent (Thermo Scientific, USA) and quantified according to the Bradford method using bovine serum albumin (BSA) as a standard (DC Protein Assay kit, Bio-Rad, France). CEA was determined from 10 μg of total protein and the results were expressed as units per mg of total protein. Data were computerized and statistically significant differences were calculated using Student's *t*-test. A *P*-value of less than 0.05 was considered statistically significant.

Alternatively, the H_2_O_2_ breakdown activity was verified using a positive catalase test (ID color Catalase; BioMérieux, France) in bacterial suspensions containing approximately 10^8^ CFU.mL^−1^ of *C. jejuni* NCTC 11168 and Bf strains.

## Results

### Proteome variations of *C. jejuni* Bf after exposure to aerobic conditions

**Figure 1:**
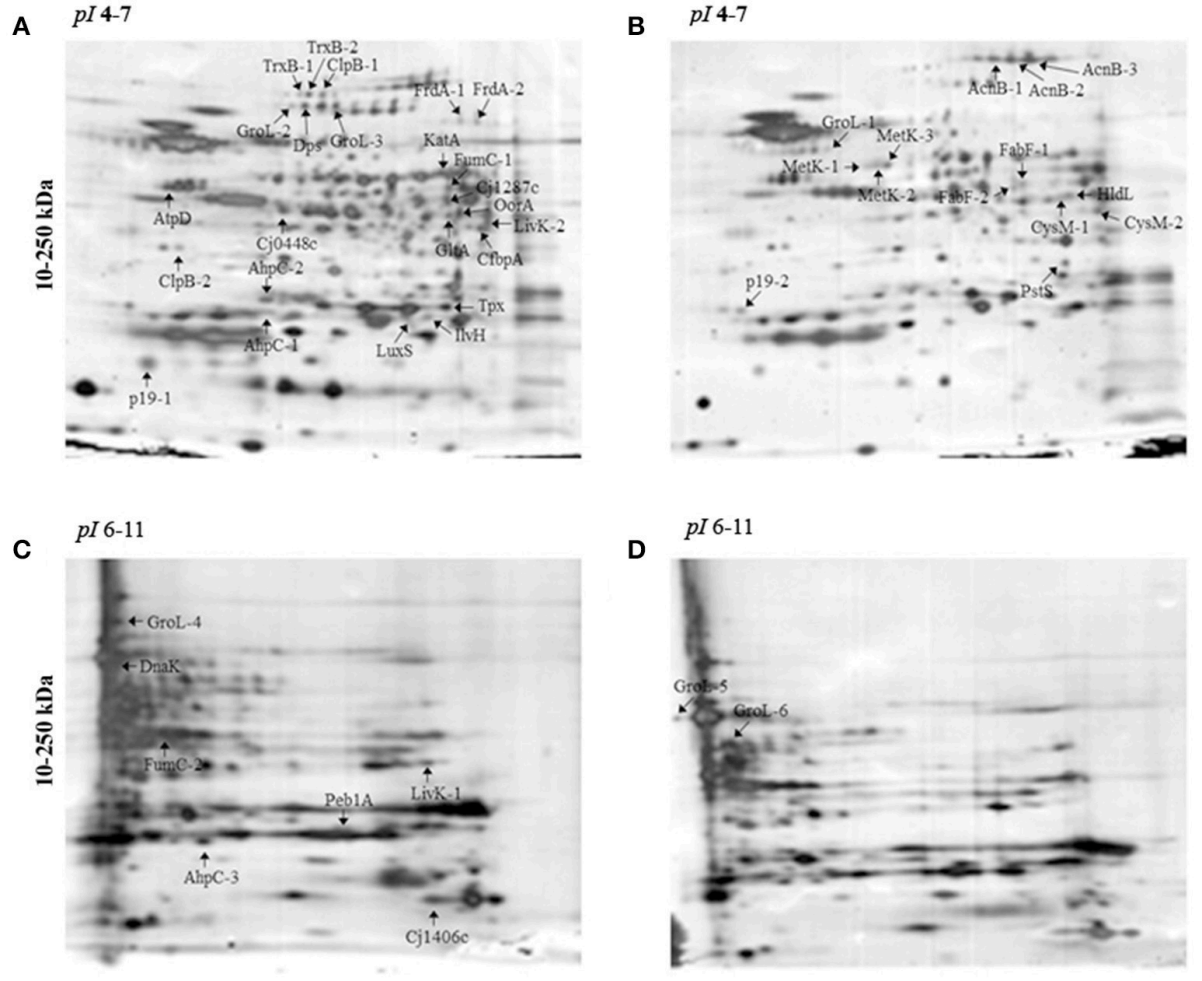
**2-DE electrophoretic profiles of *C. jejuni* Bf in aerobic (A,C) and microaerobic (B,D) conditions**. A total of 30 μg proteins was separated on IPG strips with *pI* 4-7 **(A,B)** and *pI* 6-11 **(C,D)** followed by a 4–15% gradient SDS-PAGE. Arrows indicate over-expressed proteins. Two replicates from each three independent protein extraction were performed for each condition.

Protein abundance influenced by aerobic conditions compared to microaerobic conditions in *C. jejuni* Bf was examined using 2-D gel electrophoresis. Proteins were resolved for *pI* ranges 4-7 and 6-11 (Figure 1). Overall, 47 predominant spots with a significant variation were identified (Figure 1, Table 2). Of these, 37 were detected in *pI* range 4–7 and 10 in *pI* range 6-11. Some proteins identified from several spots were considered as isoforms. Altogether, identification of 28 unique non-redundant proteins were obtained and classified into categories according to their biological function using the KEGG: Kyoto Encyclopedia of Genes and Genomes (http://www.genome.jp/kegg/) (Table 2). Based on their function, these 28 proteins, expressed differently in aerobic conditions, could be divided into 11 metabolic groups.

**Table 2:** **Identification of proteins predominantly affected in *C. jejuni* Bf under aerobic conditions**.

**Protein ID**	**Name**	**Gene**	**ANOVA^a^ (*p*-value)**	**Fold^b^**	**Theoretical MW (Da)/*pI***	**Log *E*-value^c^**	**No. of MP/TP (Pc)^d^**
**CELL PROCESS**							
**Detoxification**							
tr|Q0PBH5	Alkyl hydroperoxide reductase	*AhpC*	6.27E-03	+2.0	21.9/5.66	−110.26	25/75 (84%)
			2.03E-02	+1.8		−82.37	13/171 (65%)
			6.01E-03	+2.5		−88.39	22/46 (57%)
sp|Q59296	Catalase	*KatA*	1.08E-02	+1.7	58.29/7.74	−102.42	22/53 (46%)
sp|Q9PPE0	Probable thiol peroxidase	*Tpx*	7.25E-03	+1.7	18.40/5.13	−63.49	8/22 (47%)
tr|Q0PBZ1	Thioredoxin reductase	*TrxB*	3.92E-02	+1.5	33/5.60	−92.07	11/60 (57%)
			3.55E-03	+2.8		−65.10	9/22 (37%)
**Chaperones, chaperonins, starvation**							
sp|O69289	60 kDa chaperonin	*GroL*	7.39E-03	−1.8	57.79/5.02	−279.73	40/187 (65%)
			1.42E-02	+2.0		−33.35	6/10 (15%)
			1.07E-02	+2.1		−7.86	2/3 (6%)
			5.46E-03	+1.5		−211.64	31/50 (52%)
			4.42E-02	−2.2		−186.05	34/51 (58%)
			2.99E-02	−1.7		−227.73	34/89 (59%)
sp|Q0P891	DNA protection during starvation protein	*Dps*	2.56E-04	+2.9	17.1/5.55	−13.89	4/4 (34%)
sp|Q9PI02	Chaperone protein	*ClpB*	7.25E-03	+1.7	95.40/5.47	−110.17	27/46 (34%)
	ClpB		1.40E-02	+1.7		−62.96	16/20 (22%)
sp|O69298	Chaperone protein DnaK	*DnaK*	2.46E-02	+1.6	67.30/4.97	−270.16	48/85 (50%)
**Transport/binding proteins**							
tr|Q0P7X0	Periplasmic protein	*p19*	1.15E-03	+1.5	19.6/4.97	−97.57	16/70 (64%)
			1.05E-02	−1.5		−39.82	5/77 (50%)
tr|Q0P8K8	Putative periplasmic protein	*Cj1406c*	4.13E-02	+2.3	24.9/5.89	−17.77	8/9 (39%)
tr|Q0P8M9	Peb1A major cell-binding factor	*Peb1A*	6.10E-03	+1.5		−46.86	13/14 (45%)
tr|Q0PAQ4	Putative periplasmic phosphate binding protein	*PstS*	1.58E-04	−2.3	36/5.76	−111.37	20/70 (58%)
tr|Q0PBW4	Putative iron-uptake ABC transport system periplasmic iron-binding protein	*CfbpA*	4.08E-03	+2.1	37.4/8.97	−111.73	20/86 (57%)
tr|Q0P9N4	Branched-chain amino-acid ABC transport system periplasmic binding protein	*LivK*	1.95E-02	+2.0	39.59/8.70	−86.36	11/17 (35%)
			3.76E-02	+2.2		−86.38	11/35%
**SMALL MOLECULE METABOLISM**							
**Energy metabolism - tricarboxylic acid cycle**							
sp|O69294	Fumarate hydratase	*FumC*	1.39E-03	+1.9	50.59/6.12	−138.94	27/109 (51%)
			1.61E-03	+1.7		−156.55	30/48 (53%)
tr|Q0PBA1	Fumarate reductase flavoprotein subunit	*FrdA*	6.03E-04	+2.2	73.59/6.36	−136.96	27/71 (44%)
			2.32E-04	+2.2		−121.49	25/42 (43%)
tr|Q0P8X0	Malate oxidoreductase	*Mez*	1.82E-03	+2.4	43.90/5.78	−94.02	16/49 (40%)
tr|Q0P7U8	Citrate synthase	*GltA*	1.49E-04	+3.1	47.90/6.47	−80.33	17/42 (48%)
tr|Q0PA55	Aconitate hydratase 2	*AcnB*	6.73E-03	−1.6	92.59/6.04	−144.40	27/82 (41%)
			1.97E-02	−1.6		−196.23	32/117 (46%)
			2.30E-02	−1.5		−222.33	37/142 (50%)
tr|Q0PAY0	2-oxoglutarate:acceptor oxidoreductase	*OorA*	5.26E-03	+1.8	40.90/6.12	−74.90	17/37 (42%)
**ATP-proton motive force**							
sp|Q0PC30	ATP synthase subunit beta	*AtpD*	1.77E-02	+1.5	50.70/4.97	−137.76	25/127 (59%)
**Lipid metabolism; fatty acid biosynthesis**							
tr|Q0PB68	3-oxoacyl-[acyl-carrier-protein] synthase 2	*FabF*	3.54E-02	−1.5	42.59/5.64	−180.60	29/103 (58%)
			1.60E-02	−1.5		−74.51	12/146 (53%)
**Lipopolysaccharide biosynthesis**							
tr|Q0P9A5	ADP-L-glycero-D-manno-heptose-6-epimerase	*HldD*	3.59E-04	−1.7	35.90/6.12	−138.21	23/73 (69%)
**CENTRAL INTERMEDIARY METABOLISM**							
**Amino acid biosynthesis**							
sp|P71128	Cysteine synthase B	*CysM*	6.28E-04	−1.8	32.30/6.34	−82.93	16/67 (57%)
			6.72E-03	−2.2		−19.81	3/21 (21%)
tr|Q0PAU2	Acetolactate synthase small subunit	*IlvH*	1.48E-02	+1.8	17.3/6.75	−26.11	8/10 (52%)
**Biosynthesis of secondary metabolites**							
tr|Q0P9F8	S-adenosylmethionine synthetase	*MetK*	4.04E-02	−1.6	43.7/5.45	−103.70	19/63 (56%)
			6.50E-03	−1.9		−126.01	25/86 (55%)
			3.21E-02	−1.9		−142.72	27/102 (59%)
**BROAD REGULATORY FUNCTIONS**							
**Signal transduction**							
tr|Q0PB65	Putative MCP-type signal transduction protein	*Cj0448c*	3.17E-02	+1.6	40.40/5.58	−80.75	15/55 (47%)
**Synthesis of autoinducer 2**							
sp|Q9PN97	S-ribosylhomocysteine lyase	*LuxS*	7.25E-03	+1.7	18.10/6.19	−38.61	7/16 (50%)

a Normalized spots were compared for their abundance between aerobic and microaerobic conditions. Significant difference is indicated for each spot.

b Positive values correspond to the fold of higher abundance in aerobic conditions and negative values correspond to the fold of lower abundance in aerobic conditions for each spot.

C Each protein was identified with a mass tolerance < 20 ppm and at least two peptides.

d No. of MP/TP (Pc): Number of matched/total peptides (Protein coverage).

The main proteins found at higher abundance in aerobic conditions are involved in stress responses (AhpC, KatA, Tpx, TrxB, LuxS, GroL, Dps, ClpB, and DnaK) and carbohydrate metabolism, particularly the tricarboxylic acid (TCA) cycle (FumC, FrdA, Cj1287c, GltA, AcnB, OorA). Proteins involved in processes such as membrane transport (p19, Cj1380, CfbpA, LivK), energy metabolism (AtpD), amino acid biosynthesis (IlvH), and signal transduction (Cj0448c) were also more abundant under aerobic conditions. In contrast, proteins related to fatty acid biosynthesis (FabF), membrane transport (PstS), lipopolysaccharide biosynthesis (HldD), amino acid biosynthesis (CysM), biosynthesis of secondary metabolites (MetK), and energy metabolism through the TCA cycle (AcnB) were less abundant under aerobic conditions. An overview of enzymes differently expressed in TCA cycle and ROS detoxification during multiplication of Bf under aerobic conditions is summarized in Figure 4.

### Transcript levels of the specific genes involved in ROS detoxification in *C. jejuni* in aerobic conditions

**Figure 2:**
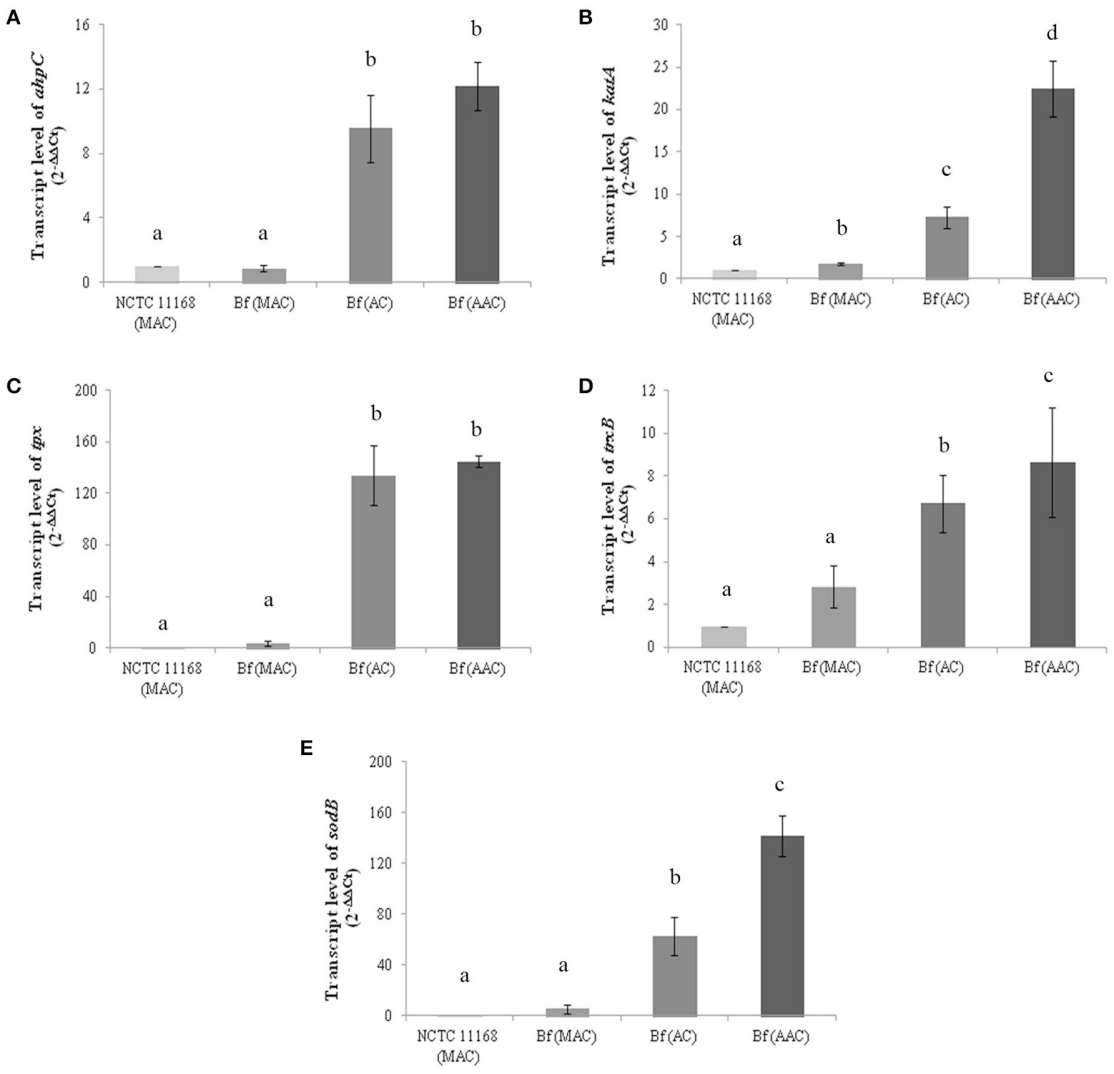
**Transcript levels of *ahpC* (A), *katA* (B), *tpx* (C), *trxB* (D) and *sodB* (E) genes in *C. jejuni* NCTC 11168 and *C. jejuni* Bf in microaerobic conditions (MAC), aerobic conditions (AC) and after acclimation to aerobic conditions (AAC)**. Transcript levels were measured by RT-qPCR and calculated using the critical threshold (ΔΔ_C*T*_) method with *rrs* gene as the internal control. Results were normalized to the gene transcription of the reference strain *C. jejuni* NCTC 11168 in microaerobic conditions. Error bars represents the standard deviation of three independent experiments. Significant differences, indicated by a different letter, were determined using the Student's *t*-test comparisons with a 5% confidence level.

AhpC, KatA, Tpx, and TrxB proteins were selected to investigate their expression patterns further at the transcriptional level. Although SodB was not detected among the more abundant proteins in aerobic conditions using 2-D electrophoresis, the transcript level of SodB was also evaluated as this protein is important for the cellular detoxification process in *C. jejuni*. The strain NCTC 11168 grown in microaerobic conditions was used as a reference strain. The transcript levels of the genes involved in ROS detoxification in *C. jejuni* Bf were evaluated in optimal growth conditions (microaerobiosis), in aerobic conditions and after aerobic acclimation. The data revealed an increase in *ahpC, katA, tpx*, and *trxB* transcript levels in *C. jejuni* Bf cells cultured in aerobic conditions or aerobically acclimated when compared to the relative transcript level of the reference strain *C. jejuni* NCTC 11168 (*P* < 0.05) (Figures 2A–D). These results indicate that the transcript level of these genes followed the same trend as the proteomic abundance changes in aerobic conditions. The expression of *sodB* also increased significantly in cells incubated in aerobic conditions (63.3-fold) and aerobically acclimated (142.1-fold) (Figure 2E).

### Effect of aerobic conditions on CEA in *C. jejuni* NCTC 11168 and *C. jejuni* Bf

**Figure 3:**
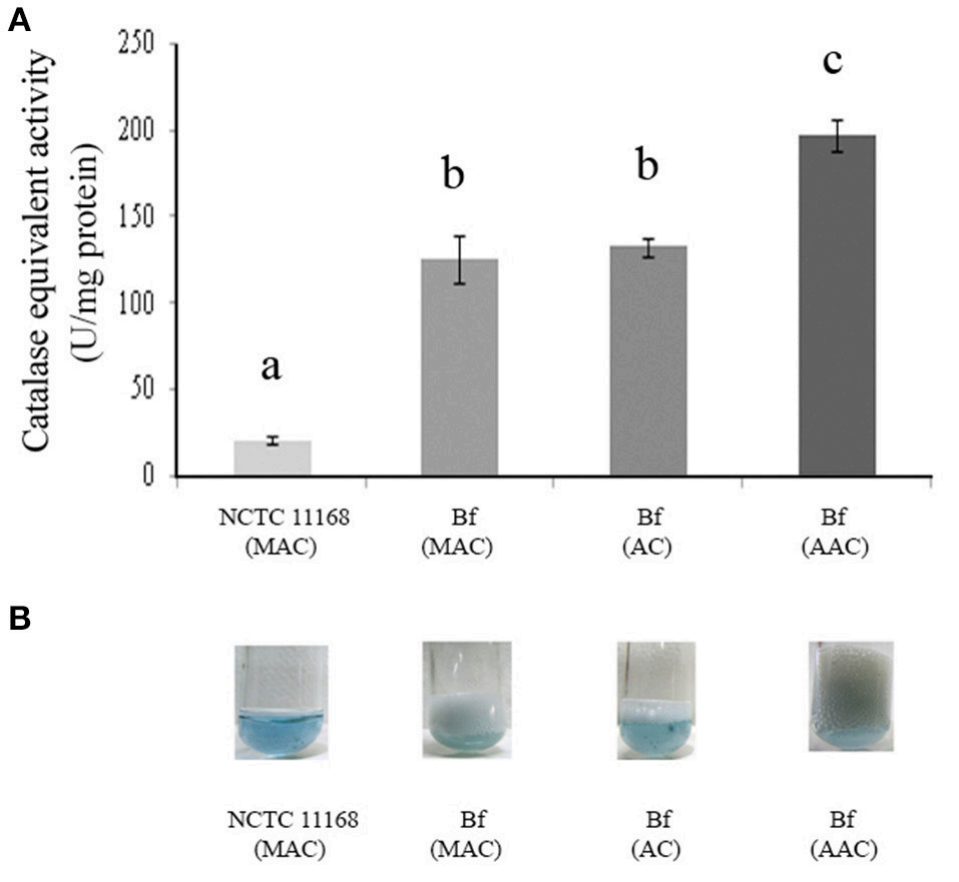
**Catalase-equivalent activity of *C. jejuni* Bf in different gaseous conditions**. H_2_O_2_cleavage was first assessed over time in the presence of calibrated quantities of bovine liver catalase and a correlation was calculated between the rate of decrease in absorbance of H_2_O_2_ and bovine liver catalase activity (*y* = 0.0153x–0.0026; *R*^2^ = 0.97). **(A)** Catalase-equivalent activity of *C. jejuni* Bf in microaerobic conditions (MAC), in aerobic conditions (AC) and after acclimation to aerobic conditions (AAC), *C. jejuni* 11168 was used as a control; **(B)** Bubble production of *C. jejuni* Bf in MAC, AC, and ACC mediated by the breakdown of H_2_O_2_ to water and dioxygen gas. *C. jejuni* 11168 was used as a control. Plotted points are the mean measurements from three independent experiments. Significant differences, indicated by a different letter, were determined using the Student's *t*-test comparisons at 5% confidence level.

H_2_O_2_ breakdown activity was quantified using the method based on the decrease in OD_240nm_ expressed as CEA. The sensitivity and accuracy of the method were assessed using known concentrations of bovine liver catalase for the calibration curve. The linear rate of H_2_O_2_ decomposition was obtained in the presence of catalase levels ranging from 0.05 U to 2.00 U (Figure 3A). The curve showed a linear slope of 0.96 (Figure 3B) indicating that the method provides an accurate estimate of the activity of catalase within the range of analysis. Cellular extracts obtained from the exponential culture of *C. jejuni* Bf grown in microaerobic conditions, in aerobic conditions or after acclimation to aerobic conditions, produced CEA corresponding to 125.93, 132.46, and 197.82 units per mg of protein, respectively, which was higher than the transcript level basis of *C. jejuni* NCTC 11168 in microaerobic conditions (20.81 units per mg of protein) (Figure 3A). In addition, these data are in agreement with the visual observations of gas production by Bf mediated by the breakdown of H_2_O_2_ to water and dioxygen gas (Figure 3B).

## Discussion

We have previously demonstrated the atypical behavior of *C. jejuni* Bf strain with its ability to grow under an ambient gaseous atmosphere unlike other strains of *C. jejuni* (Rodrigues et al., 2015). In addition, this strain showed a higher survival rate in the presence of ROS-promoting agents (paraquat (PQ) and H_2_O_2_) compared to the reference strain NCTC 11168 (Rodrigues et al., 2015). The genome sequence of *C. jejuni* Bf is being analyzed and has not yet revealed any major genetic traits related to its aerotolerance when compared to other genomes of *C. jejuni* (AN: FCEZ01000001–FCEZ01000095).

In this study, we focused on the cellular factors involved in the phenotypic response of Bf in aerobiosis. Comparison of the 2-D electrophoretic profiles between growth in aerobiosis and microaerobiosis revealed more abundant proteins involved in the oxidative stress response in *C. jejuni* including AhpC, KatA, Tpx, TrxB, LuxS, and Dps (Baillon et al., 1999; Storz and Imlay, 1999; Chelikani et al., 2004; Atack and Kelly, 2009). *C. jejuni* possesses eight major proteins involved in ROS detoxification: AhpC, SodB, KatA, Tpx, Bcp (thiol peroxidase), Dps (bacterioferritin), MsrA/B, and Cj1386 (ankyrin-containing protein involved in heme trafficking to catalase) (Pesci et al., 1994; Grant and Park, 1995; Baillon et al., 1999; Purdy et al., 1999; Ishikawa et al., 2003; Atack et al., 2008; Atack and Kelly, 2009). In addition, TrxB, LuxS, and Dps have been suggested as contributing to the oxidative stress response (Zhao et al., 2002; Ishikawa et al., 2003; He et al., 2008). The enzyme AhpC, encoded by *cj0334* in NCTC 11168, is important for aerotolerance maintenance and organic peroxide stress resistance in *C. jejuni* (Baillon et al., 1999). In *E. coli*, Ahp is composed of two subunits: the catalytic subunit AhpC and the flavoprotein AhpF, which recycles AhpC (Poole et al., 2000). In the *C. jejuni* genome, a homologs gene to *ahpF* could not be identified (Parkhill et al., 2000); however, the function of AhpF was suggested as being mediated by TrxB, like in its close relative *H. pylori* (Baker et al., 2001). Thus, the concomitant over-expression of AhpC and TrxB in the strain Bf in aerobiosis is not surprising. Defense against H_2_O_2_ cytotoxicity in *C. jejuni* is also achieved by KatA through the breakdown of two molecules of H_2_O_2_ to H_2_O and O_2_ (Storz and Imlay, 1999; Grant et al., 2003). The peroxiredoxin thiol peroxidase (Tpx), encoded by *cj0779* in NCTC 11168, confers resistance to organic hydroperoxides. The synthesis of Tpx enzyme is induced when *C. jejuni* is exposed to atmospheric concentrations of oxygen, and probably constitutes the primary defense against oxidative stress (Atack et al., 2008). Tpx, together with bacterioferritin co-migratory protein (Bcp), are considered the main defense enzymes against aerobic conditions in *C. jejuni* (Atack et al., 2008). The over-expression of AhpC, TrxB and KatA and their concomitant higher levels of transcripts in *C. jejuni* Bf in aerobiosis indicate that none of these three detoxification enzyme systems seem to be predominant to ensure the multiplication of this strain in atmospheric conditions. LuxS is described as a cellular factor playing a role in both signaling and the activated methyl cycle (Pereira et al., 2013). With the lower sensitivity of the isogenic *luxS* mutant of *C. jejuni* 81–176 to H_2_O_2_, He et al. (2008) also observed a lower transcription of the *ahpC* and *tpx* genes. Our study confirmed the importance of *luxS* in the sustainable growth under aerobiosis of *C. jejuni* Bf. Dps is a protein that can bind non-specifically to DNA, which confers protection against stress caused by peroxides (Almirón et al., 1992; Andrews et al., 2003). In addition, Dps is considered an iron storage protein (40 iron atoms per monomer) that can release iron in order to cope with iron deficiency in the environment (Andrews et al., 2003). In fact, interactions between iron and oxygen reduction residues lead to the accumulation of ^•^OH which causes widespread damage to DNA, proteins and membranes (Zhao et al., 2002). Therefore, Dps is able to minimize the effect of iron with oxidative stress to ensure survival and prevent cell death (Ishikawa et al., 2003). At the regulation level, iron homeostasis is achieved by controlling the absorption, metabolism and storage of iron in *C. jejuni* (van Vliet et al., 1999). The expression of *katA, ahpC* and *sodB* in *C. jejuni* is also iron-dependent. *katA* and *ahpC* genes are both regulated by the iron-dependent transcriptional regulators FurR and PerR (Baillon et al., 1999; van Vliet et al., 1999). KatA and SodB also require iron or iron cofactors for their catalytic activity (Pesci et al., 1994; Miller et al., 2009). Consequently, the higher abundance of Dps in aerobiosis could be correlated with the protection of *C. jejuni* against oxidative stress. This assumption is reinforced by the higher abundance of the protein CfbpA in aerobiosis, a periplasmic iron-uptake protein.

Transcriptional analyses indicated up-regulated transcript levels of the genes *katA, ahpC, sodB, tpx*, and *trxB* in *C. jejuni* Bf in aerobiosis and in aerobically acclimated cells when compared to the reference strain *C. jejuni* NCTC 11168. Interestingly, the transcript level was not significantly different between NCTC 11168 and Bf for all proteins in microaerobic conditions except for *katA*. The greater abundance of protein KatA and the simultaneously higher transcript level of its encoding gene in Bf in microaerobic conditions were confirmed by a higher CEA. This could contribute to explain the greater potential of strain Bf to adapt to aerobic conditions. This is confirmed with the increased transcript and protein levels of enzymes involved in ROS scavenging in aerobic conditions and in aerobically acclimated cells. Furthermore, the gene encoding SodB, another enzyme crucial for ROS scavenging, showed a higher transcript level in aerobiosis than in microaerobiosis. SodB is thought to provide the first line of defense against the toxic effects of reactive oxygen derivatives. This enzyme is responsible for the conversion of superoxide anions (${\text{O}}_{2}^{•-}$) to H_2_O_2_ and O_2_, thus playing a protective role against the effects of oxidative stress, as shown by several studies (Pesci et al., 1994; Purdy et al., 1999; Palyada et al., 2009). SodB was previously resolved on our *pI* range 4–7 electrophoretic gels of *C. jejuni* cytosoluble proteins (Bièche et al., 2012). The absence of detection of a higher abundance of SodB in our electrophoretic protein fingerprinting in aerobic conditions could be explained by the absence of a variation in SodB in these conditions or by a variation below the threshold of our statistical model. Altogether, these data indicate that the sub-system of oxygen detoxification is enhanced during the multiplication of strain Bf in aerobic conditions.

**Figure 4:**
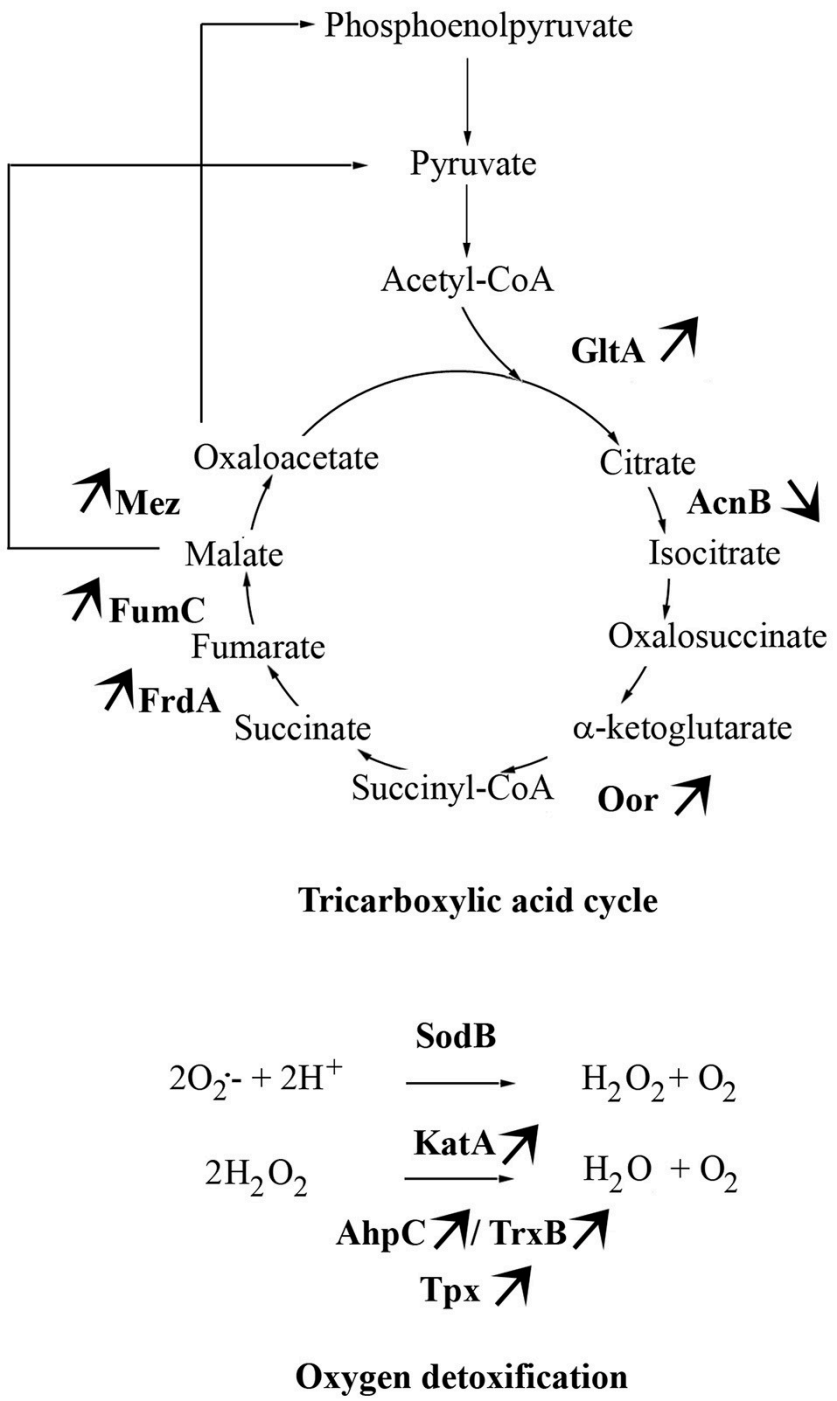
**Main metabolic pathways required by *C. jejuni* Bf in aerobic conditions**. The arrows indicate the differential abundance of enzymes when Bf is cultivated under aerobic conditions as compared to cells grown in microaerobiosis.

Concomitantly, several proteins involved in carbohydrate metabolism through the TCA cycle were more abundant in *C. jejuni* Bf under aerobic conditions. This essential metabolic pathway for all oxidative organisms provides precursors for anabolic processes and reducing factors such as NADH and FADH_2_, leading to generation of energy (Mailloux et al., 2007). *C. jejuni* is unable to use many common carbohydrates as carbon sources (Parkhill et al., 2000). So this asaccharolytic microorganism depends heavily on the TCA cycle for its carbon source through gluconeogenesis (Velayudhan and Kelly, 2002). It can uptake several TCA cycle intermediates and use them directly as nutrient sources (Stahl et al., 2012). The TCA cycle of *C. jejuni* is composed of Fe-S cluster enzymes which is not common in microaerophiles or aerophiles. Oxidation of (Fe-S) cluster of proteins has been shown to be implicated in oxidative stress susceptibility (Atack and Kelly, 2009). The Oor enzyme, which contains oxygen labile (4Fe-4S) centers, was recently demonstrated to be rapidly inactivated after exposure of cells to aerobiosis (Kendall et al., 2014). This oxygen lability was suggested as a major contributor to the microaerophily of *C. jejuni* (Kendall et al., 2014). The higher abundance of OorA in Bf under aerobic conditions tends to indicate that this protein is functional in atmospheric conditions. However, further analyses are required to determine the activity of Oor in Bf under aerobiosis. The greater abundance of most enzymes involved in the TCA cycle in Bf under aerobic conditions (Figure 4) indicates that this strain requires more carbon sources and energy in these conditions than in microaerobiosis, even though its growth rate in aerobic conditions is not higher than in microaerobic conditions (Rodrigues et al., 2015).

The replenishment of TCA cycle intermediates of this microorganism under aerobic conditions could be ensured by proteins involved in amino acid uptake detected in *C. jejuni* Bf under aerobic conditions, such as Peb1A and LivK. *C. jejuni* takes up glutamate and aspartate using an ABC transporter system encoded by the *peb* locus (*cj0919c*–*cj0922c*) (Hofreuter, 2014). Some studies have described the periplasmic permease Peb1A as a glutamate- and aspartate-binding protein (Leon-Kempis Mdel et al., 2006; Müller et al., 2007). Aspartate is considered as an efficient carbon and energy source for *C. jejuni* as it is used to feed TCA cycle intermediates by deamination to fumarate catalyzed by the aspartate ammonia lyase AspA (Guccione et al., 2008; Novik et al., 2010). Glutamate can be transformed in glutamine by glutamine synthetase GlnA (Cj0699c) or also used as substrate of the aspartate:glutamate transaminase AspB (Cj0762c) to produce aspartate and α-ketoglutarate from oxaloacetate and glutamate (Guccione et al., 2008). Moreover, glutamate catabolism and the utilization of aspartate are crucial for the metabolic fitness and growth of *C. jejuni* (Guccione et al., 2008; Hofreuter, 2014). The branched-chain amino acid ABC transporter system LIV (leucine, isoleucine, valine) of *C. jejuni* is composed of six genes encoding for two periplasmic binding proteins (LivJ and LivK), the permeases (LivH and LivM), and the cytoplasmic ATPases (LivG and LivF). Although these amino acids are not directly associated with *C. jejuni* growth (Guccione et al., 2008; Hofreuter et al., 2008), their degradation results in the synthesis of succinyl-CoA present in the TCA cycle. The higher abundance of LivK, Peb1 and IlvH tend to show that amino acid uptake and biosynthesis is important for the growth of Bf in aerobisosis.

Taken together, the atypical aerotolerance of *C. jejuni* Bf seems to result from multifactorial events ensuring an adaptive response to oxidative stress and a functional TCA cycle in aerobic conditions.

## Conclusion

Using a combined proteomic, transcriptionnal and enzymatic approach, we pinpointed the major pathways induced in the atypical strain Bf for its growth in aerobic conditions. Our findings provide new insights into the cellular mechanisms underlying the aerotolerance of *C. jejuni* Bf with the induction of an oxidative stress response, a modulation of energy production via the TCA cycle, iron uptake, amino acid uptake and factors involved in the sub-system of oxygen detoxification. This study could contribute to better understand the obligate microaerophily of *C. jejuni* and in this particular strain Bf its atypical aerotolerance. Further studies are required to explore this ability widely among *C. jejuni* strains in order to understand better the survival and contamination routes of *C. jejuni*.

## Author contributions

OT conceived the study. OT and NH and JC designed the study. RR performed the experimental work. DC performed the protein identification. RR prepared the manuscript and OT, NH, and JC contributed to the final manuscript.

### Conflict of interest statement

The authors declare that the research was conducted in the absence of any commercial or financial relationships that could be construed as a potential conflict of interest.

## Abbreviations

AAC: aerobically acclimated cells
AC: aerobic conditions
BB: bromophenol blue
BSA: bovine serum albumin
GBS: Guillian-Barré Syndrome
MAC: microaerobic conditions
MFS: Miller Fisher Syndrome
PCA: Principal Component Analysis
PQ: paraquat
ROS: reactive oxygen species
TCA: tricarboxylic acid.
